# Mechanistic investigation of Ca^2+^ alternans in human heart failure and its modulation by fibroblasts

**DOI:** 10.1371/journal.pone.0217993

**Published:** 2019-06-18

**Authors:** Maria T. Mora, Juan F. Gomez, Gregory Morley, Jose M. Ferrero, Beatriz Trenor

**Affiliations:** 1 Centro de Insvestigación e Innovación en Bioingeniería, Universitat Politècnica de València, Valencia, Spain; 2 Leon H. Charney Division of Cardiology, New York University School of Medicine, New York, New York, United States of America; University of Minnesota, UNITED STATES

## Abstract

**Background:**

Heart failure (HF) is characterized, among other factors, by a progressive loss of contractile function and by the formation of an arrhythmogenic substrate, both aspects partially related to intracellular Ca^2+^ cycling disorders. In failing hearts both electrophysiological and structural remodeling, including fibroblast proliferation, contribute to changes in Ca^2+^ handling which promote the appearance of Ca^2+^ alternans (Ca-alt). Ca-alt in turn give rise to repolarization alternans, which promote dispersion of repolarization and contribute to reentrant activity. The computational analysis of the incidence of Ca^2+^ and/or repolarization alternans under HF conditions in the presence of fibroblasts could provide a better understanding of the mechanisms leading to HF arrhythmias and contractile function disorders.

**Methods and findings:**

The goal of the present study was to investigate *in silico* the mechanisms leading to the formation of Ca-alt in failing human ventricular myocytes and tissues with disperse fibroblast distributions. The contribution of ionic currents variability to alternans formation at the cellular level was analyzed and the results show that in normal ventricular tissue, altered Ca^2+^ dynamics lead to Ca-alt, which precede APD alternans and can be aggravated by the presence of fibroblasts. Electrophysiological remodeling of failing tissue alone is sufficient to develop alternans. The incidence of alternans is reduced when fibroblasts are present in failing tissue due to significantly depressed Ca^2+^ transients. The analysis of the underlying ionic mechanisms suggests that Ca-alt are driven by Ca^2+^-handling protein and Ca^2+^ cycling dysfunctions in the junctional sarcoplasmic reticulum and that their contribution to alternans occurrence depends on the cardiac remodeling conditions and on myocyte-fibroblast interactions.

**Conclusion:**

It can thus be concluded that fibroblasts modulate the formation of Ca-alt in human ventricular tissue subjected to heart failure-related electrophysiological remodeling. Pharmacological therapies should thus consider the extent of both the electrophysiological and structural remodeling present in the failing heart.

## Introduction

Heart failure (HF) with reduced left ventricular ejection fraction is characterized by a progressive loss of contractile function and the genesis of malignant arrhythmias, leading to cardiac dysfunction and sudden cardiac death. Altered action potentials (APs), Ca^2+^ transients (CaTs) in myocytes and abnormal electrical conduction in cardiac tissue may result from the electrophysiological and structural remodeling of the failing heart, in which fibroblast proliferation has been observed [1–3].

Despite the extensive research on heterocellular connections between cardiac myocytes and non-excitable cells, the existence of this heterocellular coupling *in vivo* is still controversial [4]. The first experimental evidence was found in isolated cells and cultures in animal models and showed functional gap junctions between fibroblasts and myocytes, allowing electrical propagation [5–8]. More recent studies have demonstrated electrical coupling between scar tissue and the surrounding myocardium in live injured hearts [9–11]. These electrotonic interactions affect myocyte impulse conduction and electrophysiological activity [12–15] and increase the arrhythmogenic consequences [16,17]. Although numerous studies have used computer simulations of cardiac tissue with fibroblast coupling to investigate the origin of reentrant activity [18,19], including our previous work on failing tissue [20], the role of beat-to-beat alternans has not yet been considered.

Mechanoelectrical alternans, i.e. T-wave and contractile beat-to-beat oscillations, have been observed in HF patients [21]. Electrical alternans are linked to arrhythmogenesis because of dispersion of refractoriness and have become a risk indicator of sudden cardiac death [22], in addition to the ventricular dysfunction resulting from mechanical alternans. Cardiac alternans manifest as AP and Ca^2+^ cycling fluctuations in myocytes due to the fact that the membrane voltage and Ca^2+^ processes are coupled. However, the underlying mechanisms leading to alternans have not yet been fully identified and the feedback nature of coupling between voltage and Ca^2+^ dynamics complicates the task. Among the proposed mechanisms, voltage-driven Ca^2+^-mediated alternans and the contribution of both systems [23–27] have been suggested.

The most extensively developed theory is that alternans are induced by Ca^2+^ cycling instabilities, by which an altered sarcoplasmic reticulum (SR) Ca^2+^ release process through the ryanodine receptor (RyR) impairs Ca^2+^ dynamics and initiates alternans [28,29]. Fluctuations in the SR Ca^2+^ content [30] have suggested an impairment beween Ca^2+^ uptake and release mechanisms. Computational studies have analyzed the role of the different Ca^2+^-handling proteins and their interactions that contribute to Ca^2+^ alternans [31,32]. In agreement with computational results, SERCA overexpression has been shown to supress cellular alternans [33].

Since the normal heart can develop alternans at rapid heart rates, the vast majority of experimental and computational studies on cellular alternans [28,31,34] have been performed on non-failing myocytes or without considering all the main HF features, such as electrophysiological remodeling, intercellular decoupling and fibroblast coupling. These changes in the cardiac tissue modify myocyte electrophysiology and could alter the susceptibility to alternans. We consider that the passive characteristics of the tissue also play a role in generating alternans. Discordant alternans can produce spatial repolarization gradients that may generate electrical reentries leading to ventricular fibrillation [35], so that it is crucial to determine the causes and susceptability to spatially discordant versus concordant alternans.

The aim of this study was thus to characterize the onset of alternans in HF under the influence of disperse fibroblasts using a virtual two-dimensional human model. In the first part of the study, we used a mathematical model of a single human ventricular myocyte to study its bioelectrical activity, including Ca^2+^ dynamics in health and during HF, coupled or uncoupled to fibroblasts. Simulations were performed to determine the influence of pacing frequency on the alternans occurrence threshold. In the second part we simulated the activity of a virtual ventricular tissue with normal and failing myocyte populations, with or without inserted fibroblasts. We built populations of models by imposing variability to ion channels and pumps. The biological variability represented in the different models can reveal the most favorable conditions that induce Ca^2+^ beat-to-beat alternans and, by means of the Ca^2+^-driven theory, the important role of Ca^2+^-handling proteins in modifying Ca^2+^ dynamics.

## Methods

### Cellular simulations

Cardiac tissue consists of interconnected cardiomyocytes and other non-muscular cells, such as fibroblasts, which might establish electrical connections with myocytes through gap junctions. The role of fibroblast-myocyte interactions at the onset of alternans was first studied in single-cell simulations and compared to two coupled myocytes to identify the key parameters responsible for beat-to-beat oscillations, as well as the impact of HF. The reduction of the variables at the cellular level made it possible to reduce the computational time in the 2D study. The second study was essential in order to understand the origin, features and effects of alternans, such as arrhythmogenic spatial discordance.

The O’Hara et al. [36] human AP model (ORd) was used to simulate the electrophysiological activity of endocardial myocytes. As in our previous work [37], small changes were made to the sodium current (I_Na_) in the model to improve electrical conduction and update the late component to the most recent experimental evidence. The active fibroblast model developed by MacCannell et al. [38] was coupled to myocytes to analyze fibroblast-myocyte interactions. Myocyte-fibroblast coupling was performed taking into account an individual gap junction conductance (G_gap_) of 3 nS, within the experimental range [8], which was increased five-fold to establish a 1:5 ratio between cells, accounting for an increased degree of disperse fibroblasts. Although cell-to-cell coupling between myocytes is stronger, the experimental measurements in myocyte pairs span a wide range [39,40], so that the G_gap_ was set to an intermediate value of 1.3 μS. HF conditions, i.e. electrophysiological myocyte remodeling, were also simulated. In HF, myocyte-myocyte coupling was reduced by 50%.

Electrophysiological remodeling in HF was simulated by applying a scale factor to the maximal conductances of the selected ionic channels (Table 1), which represents an upregulation or downregulation of protein expression and/or function obtained from experimental human myocytes when possible, as detailed in Gomez et al. [41].

**Table 1:** Electrophysiological remodeling in HF applied to the basic ORd model.

ORd model parameter	Upregulation/ Downregulation in HF remodeling
I_NaL_	+80%
τ_hL_	+80%
I_to_	-60%
I_K1_	-32%
I_NaK_	-30%
I_NCX_	+75%
CaMKa	+50%
J_SERCA_	-50%
J_leak_	+30%
K_rel,Ca_	-20%

The modified parameters in heart failure (HF) are: the late Na^+^ current (I_NaL_), the inactivation time constant of I_NaL_ (τ_hL_), the transient outward current (I_to_), the inward rectifier K^+^ current (I_K1_), the Na^+^/K^+^ pump current (I_NaK_), the Na^+^/Ca^2+^ exchanger (I_NCX_), the active fraction of the Ca^+2^ calmodulin-dependent protein kinase II (CaMKa), the sarcoplasmic reticulum (SR) Ca^2+^ pump (J_SERCA_), the SR Ca^2+^ leak (J_leak_), and the sensitivity to [Ca^2+^]_JSR_ of the ryanodine receptors (Ca^2+^ sensitivity of J_rel,∞_, called K_rel,Ca_).

Alternans typically occur at fast pacing rates. The pacing cycle length (PCL) threshold was determined by computing a restitution curve starting at a PCL of 1000 ms and reducing the cycle length until loss of capture was observed. Each simulation was run until steady-state was reached. The last two APs and CaTs were used to measure AP duration from maximal upstroke to 90% of repolarization (APD_90_) and the maximum intracellular [Ca^2+^], respectively.

After determining pacing threshold in the baseline models, populations of models were built and simulations were carried out at a fixed low PCL in which alternans tend to occur. Populations of models consisted of different cellular models built after varying the selected parameters related to ionic currents of the baseline model, mimicking biological inter-individual variability. We modified 13 maximal ion channel conductances and fluxes simultaneously using random scale factors obtained from a normal distribution of mean equal to 1 and standard deviation (σ) equal to 0.15, so that 95% (±2σ) of the parameters varied in the range ±30% of their baseline values. The selected variables affect the conductances of the fast Na^+^ current (I_Na_), late Na^+^ current (I_NaL_), transient outward K^+^ current (I_to_), L-type Ca^2+^ current (I_CaL_), rapid delayed rectifier K^+^ current (I_Kr_), slow delayed rectifier K^+^ current (I_Ks_), inward rectifier K^+^ current (I_K1_), Na^+^/ K^+^ ATPase current (I_NaK_), Na^+^/Ca^2+^ exchange current (I_NCX_), SR Ca^2+^ uptake via SERCA pump (J_SERCA_), SR Ca^2+^ release flux via RyR (J_rel_), SR Ca^2+^ leak (J_leak_), and Na^+^ background current (I_Nab_). Four basic cellular models were considered with different baseline conditions and/or coupling to build the populations: normal myocyte coupled to normal myocyte (N-N), normal myocyte coupled to fibroblast (N-Fb), failing myocyte coupled to failing myocyte (HF-HF), and failing myocyte coupled to fibroblast (HF-Fb). Populations of 300 different human AP models were analyzed and compared to study the role of ion channel variation in alternans generation.

Alternans were measured in AP and CaT waveforms, as shown in Eqs (1) and (2), but the final model classification criterion was the Ca^2+^ alternans ratio [34], i.e. the relative differences between the small and large CaT amplitude (CaTA), since these were more evident than APD variations. While 0 indicated no alternation, models with Ca-alt > 0.1 were considered as having alternans.

$$Ca-alt\;=\;1-\frac{{CaTA}_{small}}{{CaTA}_{large}}$$ (1)

APD alternans (APD-alt) were also calculated as the APD_90_ difference between two consecutive beats.

$$APD-alt\;=\;{APD}_{90\;Long}-{APD}_{90\;Short}$$ (2)

The models were classified into two groups according to whether or not the CaTs presented alternation. The analysis of the differences in parameter scaling factors between both groups determined the mechanistic contribution of ionic parameters to alternans generation. Statistical differences between groups were determined by the Wilcoxon rank-sum test (p < 0.05). Simulations and data analysis were performed on MATLAB (R2017b, Mathworks).

### Tissue simulations

Electrical propagation was simulated in a set of two-dimensional strands (15 mm along the x-axis and 2 mm along the y-axis) formed by myocytes and fibroblasts. Longitudinal fiber orientation was assigned along the x-axis. The tissue was isotropic, i.e. longitudinal and transversal conductivities were identical. The domain was discretized at a spatial resolution of 0.1 mm, which provided 3000 squared elements and 3171 nodes within the tissue, where the cellular model was computed. The time-step for the numerical method was set to 0.01 ms to ensure numerical convergence.

**Fig 1:**
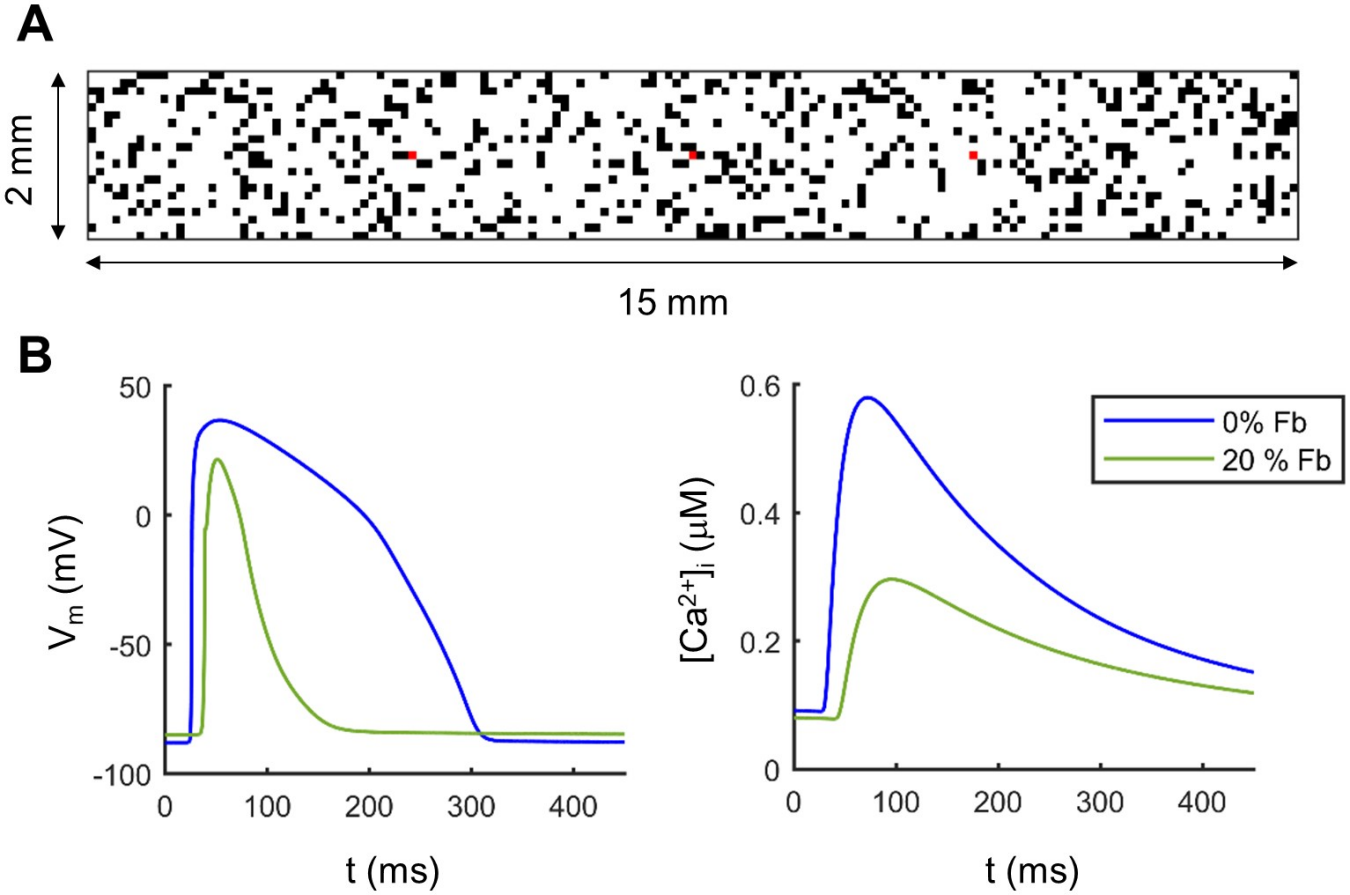
Electrophysiology in cardiac tissue simulations. A) Distribution of fibroblasts (20%; black squares) in a tissue of myocytes. Each square represents a node of the mesh and measurement nodes are marked in red. B) Action potential (AP) and calcium transient (CaT) waveforms obtained in the central node of the tissue without fibroblasts by stimulating at 1.25 Hz (blue traces) and with 20% of fibroblasts (green traces).

20% of disperse fibroblasts were considered in half of the cases and were randomly intercalated among the myocytes, as shown in Fig 1A, i.e. 20% of the mesh nodes were solved with the fibroblast model. Electrotonic coupling between myocytes and fibroblasts was weaker than myocyte-myocyte coupling. A myocyte-myocyte diffusion coefficient (D) of 0.0024 cm^2^/ms was used, which yielded a conduction velocity (CV) of 0.7 m/s along the fibers in normal conditions [42]. To simulate intercellular uncoupling, diffusion between myocytes was reduced by 50% in HF (D = 0.0012 cm^2^/ms) and 3-fold for the fibroblast-myocyte and fibroblast-fibroblast coupling (D = 0.0008 cm^2^/ms). The assignment of the diffusion coefficient to each element was chosen as in Gomez et al. [20].

Stimulation was applied to the left (short) side of the tissue and APs and CaTs were recorded in three different myocytes after 400 beats to ensure steady-state waveforms and alternans. We used the critical PCL obtained in the first part of the study (couple single-cells simulations) at which alternans appeared. Initial simulations at a basal PCL of 800 ms were conducted in tissues with different configurations to ensure that the results were independent of variations in fibroblast distribution (Fig 1B).

To reduce the computational cost of these tissue simulations, instead of considering a large population, as in the coupled single cells, we considered a subpopulation of models by varying the four most crucial factors that led to cellular alternans in the coupled single-cell simulations in the first part of the study, assuming that this subpopulation could be used to elucidate the onset of arrhythmogenic events and also the different behavior of failing and fibrotic tissue.

The most important parameters that contributed to alternans were selected from the coupled single-cell populations of models. The cardiac tissue subpopulation involved varying parameters by ±30% of their maximum value and accounted for all the possible combinations (3^4^ = 81). As in the coupled single-cell populations, we classified models into two groups: alternans (Ca-alt > 0.1) and no alternans, and calculated APD-alt. The magnitude of alternans and the spatial distribution of alternans along the tissue were also taken into account.

The simulations were performed on ELVIRA software [43], which is based on the finite element method to solve the monodomain equation by the operator splitting technique [20,44].

## Results

### Coupled single-cell simulations

#### Restitution curve

The initial simulations in coupled single cells provided a preliminary overview of the electrophysiological behavior in myocytes under pathological conditions and fast pacing rates to force beat-to-beat alternation. The following models were compared: N-N, N-Fb, HF-HF, and HF-Fb.

**Fig 2:**
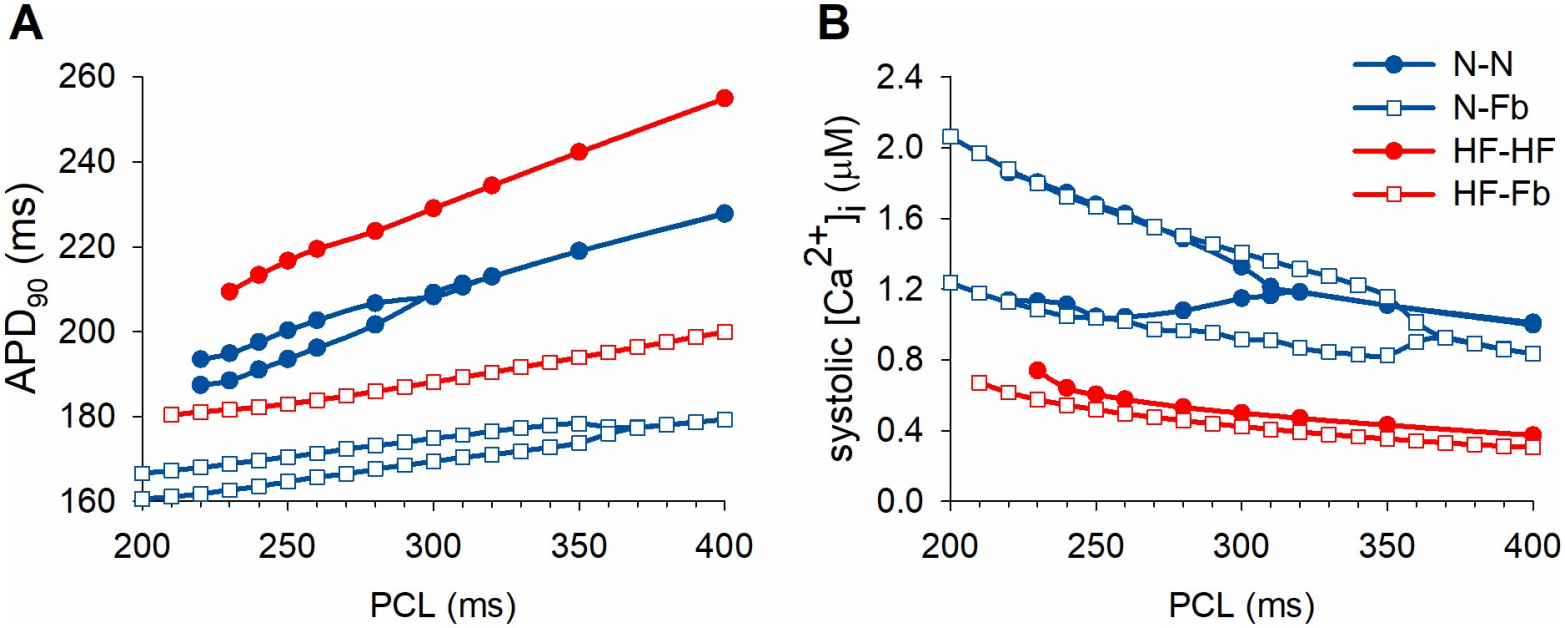
Restitution curves of baseline coupled single-cell models. A) Action potential duration (APD_90_) and B) Systolic [Ca^2+^]_i_. Comparison of electrophysiological indicators and alternans onset in myocyte-myocyte (solid circles) and myocyte-fibroblast (1:5 ratio) aggregations (empty squares) under normal (blue) and failing (red) conditions.The four traces are: normal myocyte coupled to normal myocyte (N-N), normal myocyte coupled to fibroblasts (N-Fb), failing myocyte coupled to failing myocyte (HF-HF), and failing myocyte coupled to fibroblasts (HF-Fb).

Fig 2 shows the restitution curves of APD_90_ and systolic Ca^2+^ of the four baseline models. These data indicate that an increase in heart rate leads to fluctuations in APD_90_ in consecutive beats in N-N, which are concomitant with Ca-alt. The large magnitude of Ca-alt is more evident than APD-alt. Fig 2 also shows the different alternans thresholds, according to the simulated conditions of the baseline models. While the CaT of N-N begins to alternate at a PCL of 300 ms, the electrical interactions between myocytes and fibroblasts in N-Fb increase this value to 360 ms. Myocyte-myocyte interactions seemed to slightly affect the bidirectional coupling between APD_90_ and Ca^2+^alternans, as there were small Ca-alt not translated into APD-alt.

HF-HF myocytes, with changes in APD_90_ and CaT amplitude, do not show alternans at fast pacing rates and end up in loss of capture at an early PCL equal to 250 ms. The HF-Fb model presents a lower APD_90_ and loss of capture is delayed. Although it is difficult to compare the slope of the restitution curves for the four models at high frequencies, when the PCL is longer, HF-HF shows a steeper slope than N-N and fibroblast coupling contributes to reducing the slope.

To clarify the origin of alternans, an AP clamp test was simulated (results not shown). When forcing fixed APs, Ca^2+^ dynamics alternans were not eliminated, while when fixing [Ca^2+^]_i_, [Ca^2+^]_JSR_ or J_rel_, all the alternans types disappeared, revealing a Ca^2+^-driven mechanism. Positive coupling between Ca^2+^and APD alternans was also observed, as the large (small) CaT was always in phase with the long (short) APD. NCX was the mechanism coupling between Ca-alt and APD-alt, by generating a higher inward current during the large CaT to remove Ca^2+^, which prolonged APD and suppressed the small reducing effects of an inward Ca^2+^-inactivated I_CaL_.

#### Populations of models

**Fig 3:**
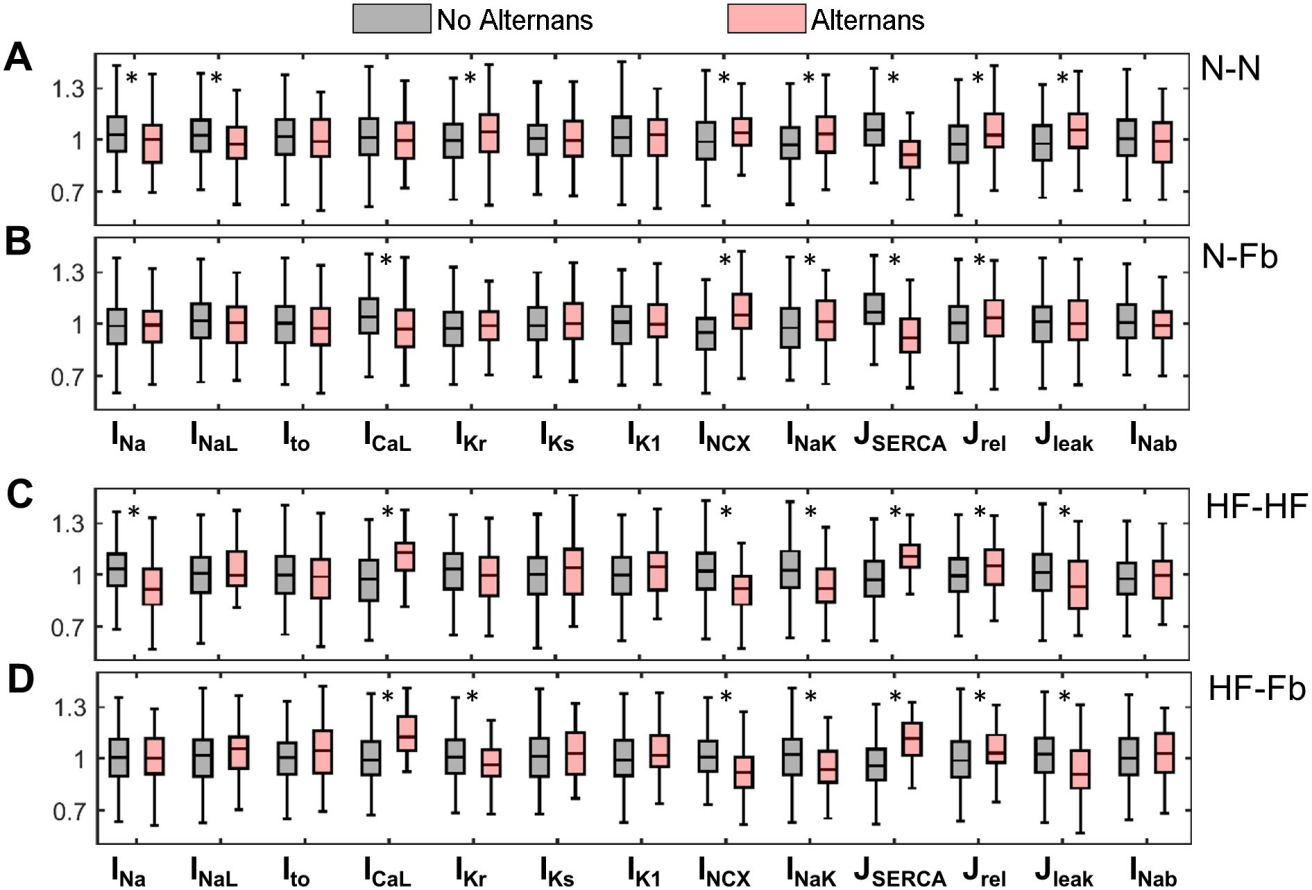
Parameter distribution according to alternans occurrence in coupled single-cell populations. The four populations are: A) normal myocyte coupled to normal myocyte (N-N), B) normal myocyte coupled to fibroblasts (N-Fb), C) failing myocyte coupled to failing myocyte (HF-HF), and D) failing myocyte coupled to fibroblasts (HF-Fb). Parameter values are represented by the scaling factors applied to the baseline models. A value of p < 0.05 (*) in the medians was considered statistically significant.

The four baseline models described above were used to build populations of cells by introducing natural variability into the electrophysiological variables. Small inter-individual differences can have remarkable effects on alternans development and may help to understand their origin. The boxplots in Fig 3 show the distribution of ionic conductances after classifying the models into the non-alternans or alternans groups. With scale factors in the range of 0.3–1.3, significant differences were found in I_Na_, I_NaL_, I_Kr_, NCX, I_NaK_, SERCA, J_rel_ and J_leak_ for the N-N population. In N-Fb models, the effect of I_CaL_was also significant, as well as that of NCX, I_NaK_, SERCA and J_rel_. In the HF setting (HF-HF and HF-Fb), although there was agreement between the main mechanisms that contribute to alternans, unlike healthy myocytes, the failing myocytes in the alternans group presented the inverse modulation of Ca^+2^-related parameters, regardless of fibroblast content. While high I_CaL_ and SERCA values and small NCX and J_leak_ values facilitated alternans in the failing populations, normal populations showed the opposite behaviour. As an exception, the impact of J_rel_ was consistent in all the populations because the higher Ca^+2^ release induced alternans.

### Cardiac tissue simulations

#### Two-dimensional populations of models

Cardiac tissue myocyte simulations provide more reliable information than coupled single-cell simulations, due to electrical propagation. Four tissue configurations were considered: normal myocytes (N-tissue), failing myocytes (HF-tissue), normal myocytes and fibroblasts (N-Fb-tissue), and failing myocytes and fibroblasts (HF-Fb-tissue). Ionic parameters in myocytes (SERCA, I_CaL_, NCX and I_NaK_) were varied to generate sets of tissue prone to alternans, on the basis of the mechanisms observed in the coupled single-cell populations. The variation of the four most important parameters related to alternans generation yielded 81 tissue models for each configuration (see Methods). The numbers of alternans cases in the different tissues are given in Table 2.

**Table 2:** Alternans occurrence in four subpopulations of cardiac tissue.

	N	HF
**without fibroblasts**	37%	43 (65) %
**20% fibroblasts**	52%	30%

There were four tissue configurations: healthy myocytes (N) or myocytes with heart failure remodeling (HF), combined with or without disperse fibroblasts (20% of nodes). The pacing cycle length was set to 300 ms. Four electrophysiological parameters were modified (I_CaL_, SERCA, NCX and I_NaK_). Alternans were Ca-alt > 0.1. Values in brackets consider 2:1 block of action potentials as alternans.

Although the magnitude of alternans can be different, accounting for the central myocytes exhibiting Ca-alt > 0.1, the results obtained highlight the major incidence of alternans in HF-tissue (including loss of AP capture). The effect of disperse fibroblasts in cardiac tissue increases alternans events in N-Fb-tissue, while it reduces the occurrence in HF-Fb-tissue. In HF-tissue, many simulations end up in one AP for every two consecutive stimulations (loss of capture), which were considered as a special case of alternans, although the origin could be different. This 2:1 block activity was not observed in any of the other cases.

**Fig 4:**
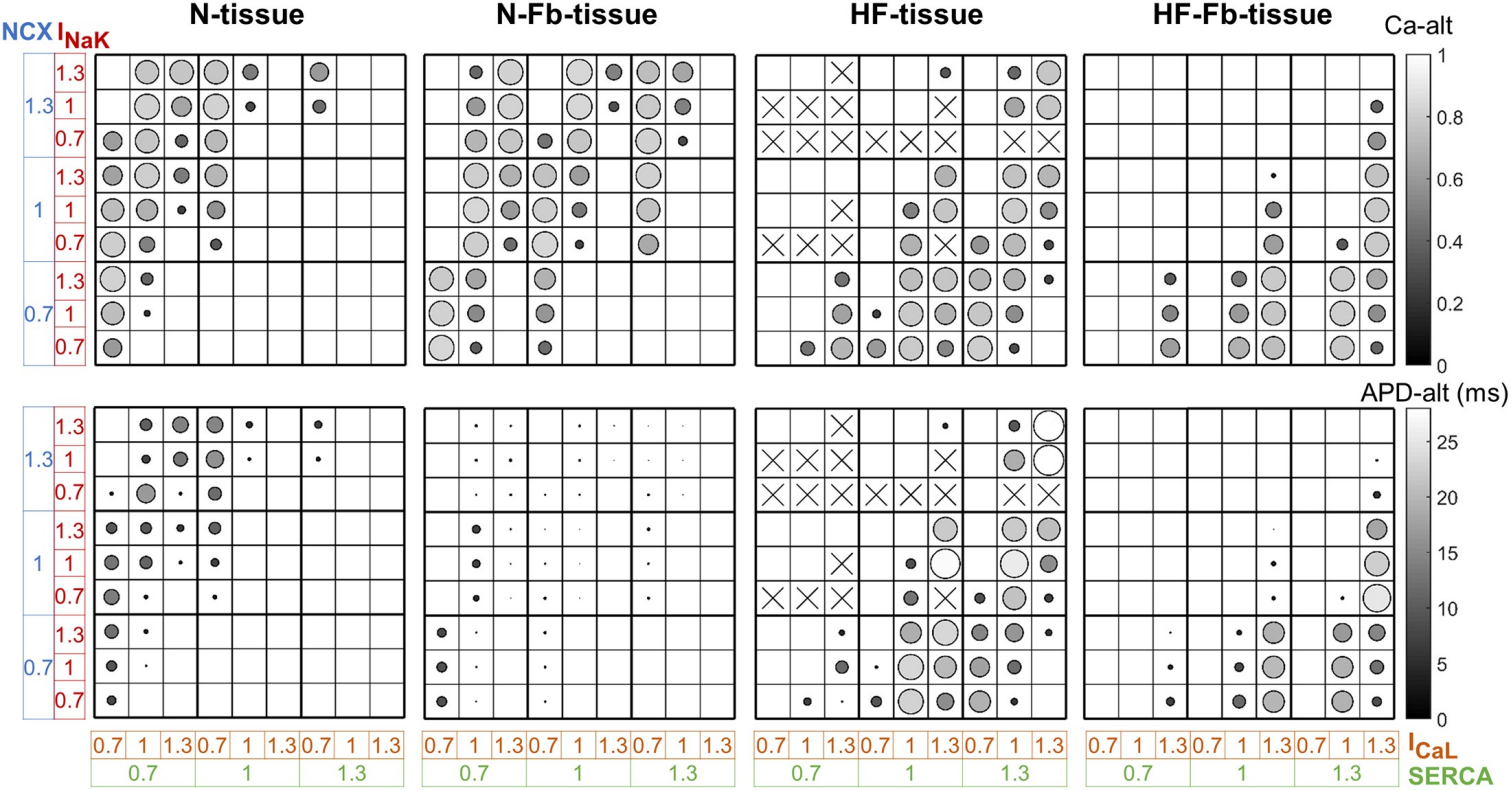
Ca^2+^ and APD alternans (Ca-alt and APD-alt) in tissues. Ca-alt (upper panels) and APD-alt (lower panels) of every model variant is classified according to the parameter values that lead to those results. The four tissue configurations are: normal myocytes (N-tissue), failing myocytes (HF-tissue), normal myocytes and fibroblasts (N-Fb-tissue), and failing myocytes and fibroblasts (HF-Fb-tissue). Alternans magnitude is proportional to circle size and gray scale. Crosses represent 2:1 block.

Fig 4 contains a graph showing Ca-alt and APD-alt values in circles and a gray scale of all the tissue sets arranged in 9x9 matrixes. The corresponding SERCA, I_CaL_, NCX, and I_NaK_ scaling factors specific to each myocyte variant classify the models in the matrix squares. In N-tissue, alternans mainly occur with reduced SERCA and I_CaL_ and high NCX. While Ca-alt can rise to values of up to 0.8 in amplitude ratio, the maximum APD_90_ variation between consecutive beats is only 15 ms. The same mechanisms induce alternans in N-Fb-tissue, but most APD-alt become negligible despite the greater incidence of Ca-alt. When the tissue included failing myocytes, the ionic modulation inducing Ca-alt of similar magnitude is different to the one observed in healthy myocytes. In HF-tissue, the repolarization alternans values are higher (up to 30 ms), although they are still of considerable magnitude in HF-Fb-tissue. The analysis of the loss of capture of AP in HF-tissue (shown by a cross) reveals that increased NCX, together with low SERCA and low I_NaK_ activity lead to this outcome.

**Fig 5:**
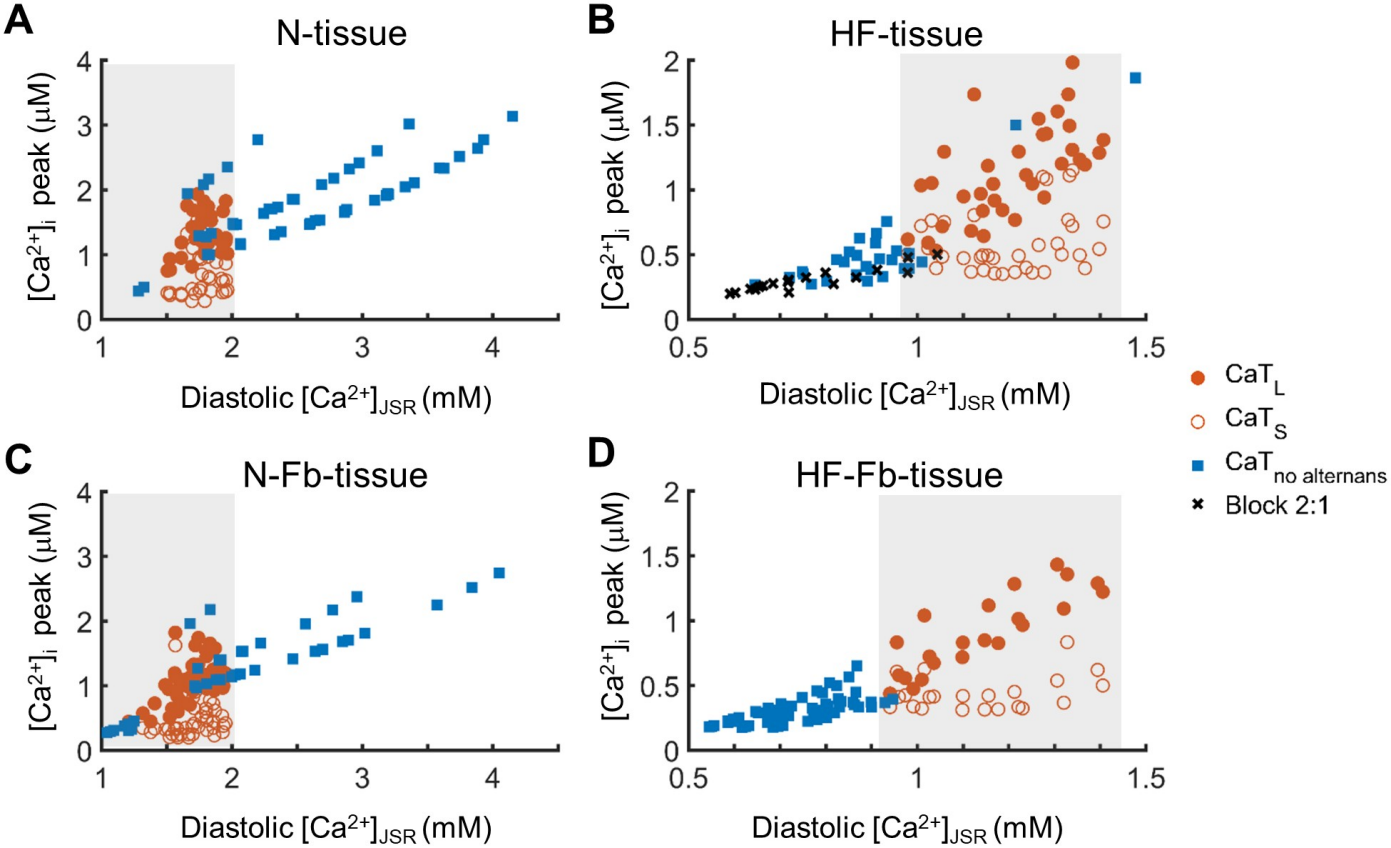
Relation between SR Ca^2+^ content, Ca^2+^ peak and alternans. The CaT peak (systolic [Ca^2+^]_i_) is related to the the maximum junctional sarcoplasmic reticulum Ca^2+^content (diastolic [Ca^2+^]_JSR_) of the myocyte. Comparison of 4 populations obtained in cardiac tissue: A) normal myocytes (N-tissue), B) failing myocytes (HF-tissue), C) normal myocytes and fibroblasts (N-Fb-tissue), and D) failing myocytes and fibroblasts (HF-Fb-tissue). Shaded areas highlight regions where alternans cases prevail.

Differences were found in CaT amplitude of the alternans and no alternans groups of the different populations. Since intracellular Ca^2+^ is linked to the amount of Ca^2+^ stored and released from the SR, the systolic Ca^2+^ peak was compared to maximum diastolic [Ca^2+^]_JSR_ (see Fig 5). The linear correlation confirmed that cytosolic and sarcoplasmic Ca^2+^ content are related and also indicated that Ca-alt occurs within a specific range of [Ca^2+^]_JSR_ values. This range (shaded area) was very similar for all four groups, but the normal myocytes (N-tissue and N-Fb-tissue) had the lowest values of [Ca^2+^], while HF (HF-tissue and HF-Fb-tissue) had the highest Ca^2+^ levels of the interval covered by the different models.

#### Spatial and mechanical discordance

**Fig 6:**
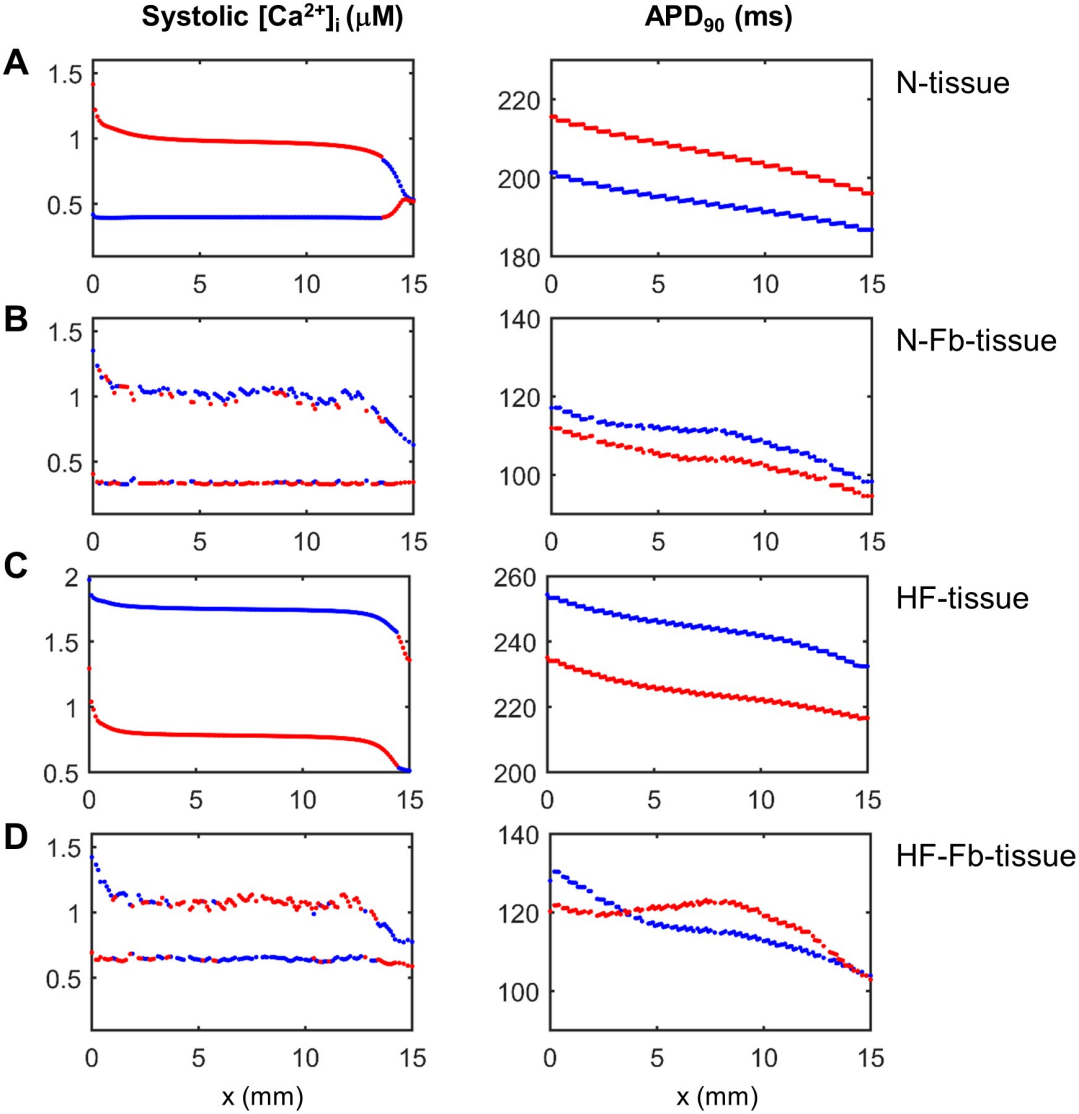
Spatial distribution of Ca^2+^ peak and APD_90_ in tissues. Node values (myocytes) are given for the central fiber of the strand for 2 consecutive beats (red and blue). The four tissue configurations are: A) normal myocytes (N-tissue), B) normal myocytes with fibroblasts (N-Fb-tissue), C) failing myocytes (HF-tissue), and D) failing myocytes with fibroblasts (HF-Fb-tissue). Y-axis has the same scale in Ca^2+^ and APD graphs, respectively but values can vary for the sake of clarity.

As cardiac alternans can become arrhythmogenic and the impact depends on the spatial distribution of the electrical fluctuations, we analyzed the evolution of alternans along the strand. Fig 6 shows APD_90_ and [Ca^2+^]_i_ peak for two consecutive beats (blue for even and red for odd beats) in a representative case from each population, quantified in the central nodes of the tissue along the longitudinal axis (x). APD-alt patterns are long-short (L-S) and Ca-alt patterns are large-small (L-S). When a long APD is concomitant with a large CaT (same color) there is positive V-Ca coupling or electromechanical concordance, otherwise there is electromechanical discordance. Consecutive dots with the same color show that alternans between adjacent myocytes are in phase (spatially corcordant), while changes in color indicate a change in alternans patterns between consecutive myocytes (spatially discordant). Spatial concordance in APD alternans is predominant in the four populations because all the myocytes are in phase following the same pattern in APs. This can be seen in both N-tissue (Fig 6A) and HF-tissue (Fig 6C), where spatial concordance is found in APD and Ca alternans.The CV in N-tissue was 0.65 m/s and the characteristic cellular uncoupling in HF reduced it to 0.4 m/s in HF-tissue but the alternans electrical propagation does not seem to be affected. In some cases, discordance can appear in Ca^2+^ dynamics in normal tissue or in both APD and Ca^2+^ activity in failing tissue. Of all the N-tissue simulated, only 13% of tissue variants with Ca-alt are spatially discordant, while in N-Fb-tissue all the Ca-alt become discordant. In these tissues with fibroblasts, APD_90_ alternans are concordant, so that electromechanical discordance occurs (Fig 6B). However, in failing tissues, discordant Ca-alt do not increase significantly from HF-tissue (7.5% alternans) to HF-Fb-tissue (12.5% alternans). What distinguishes HF-Fb-tissue from N-Fb-tissue is that spatial discordance occurs in both Ca-alt and APD-alt and is less chaotic (Fig 6D), which could be related to a slower CV (0.34 m/s vs 0.44 m/s in normal tissue with fibroblasts).

In addition to spatial differences, Fig 6 shows maximum and minimum APD_90_ and systolic [Ca^2+^]_i_ values. Although these values also depend on the modulation of ionic parameters, the figures show that although there are differences in APD_90_, small and large CaTs from different myocytes with alternating activity can become similar in magnitude. The APD_90_ prolongation characteristic of HF appears in HF-tissue and affects APD-alt, but there is also a significant reduction in APD_90_ in fibrotic tissues (N-Fb-tissue and HF-Fb-tissue) due to myocyte-fibroblast interactions.

**Fig 7:**
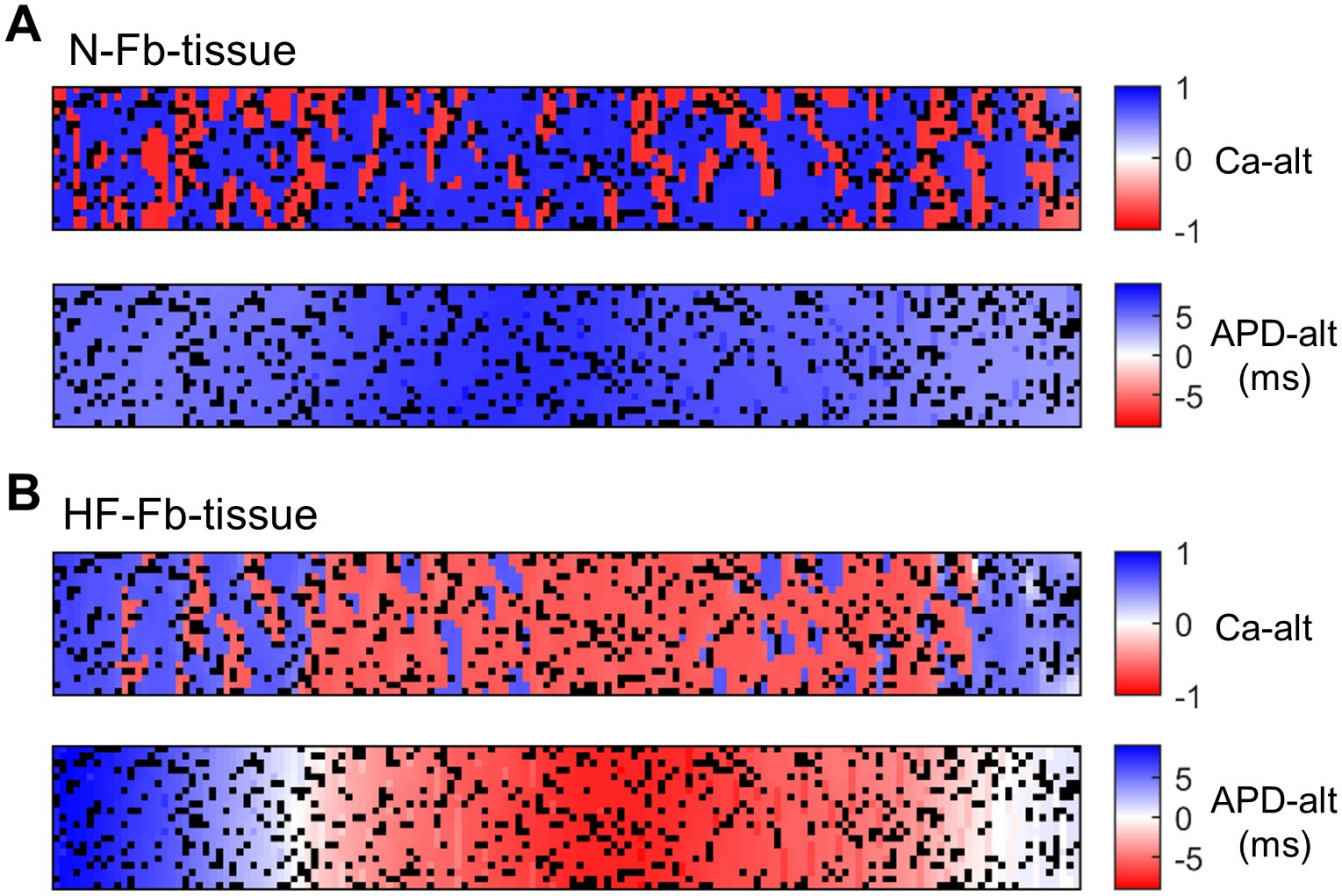
Spatial concordance of alternans (Ca-alt and APD-alt) along the tissue. A) Tissue with normal myocytes and fibroblasts (N-Fb-tissue), and B) tissue with failing myocytes and fibroblasts (HF-Fb-tissue). Opposite signs (red vs blue) indicate out-of-phase alternans. Fibroblasts are represented in black.

Since fibroblasts seem to contribute to a more chaotic spatial distribution of Ca-alt, the whole tissue is represented in Fig 7. Black squares indicate the specific location of disperse fibroblasts and the color scale represents alternans magnitude. Alternans of opposite signs, i.e. L-S and S-L, in both CaT (upper panels) and APD (lower panels) are shown in red and blue. As in Figs 6B and 7A shows that in N-Fb-tissue the spatially discordant Ca-alt affect the entire tissue but are not translated to APD-alt. Discordant areas vary between tissue variants of the N-Fb-tissue population, but all show a similar chaotic Ca-alt distribution (results not shown) with no patterns around the fibroblast locations. When spatial Ca-alt occur in HF-Fb-tissue (Fig 7B), they are confined to certain tissue regions in which APD-alt also appear, enhancing electromechanical concordance. Fig 7B also shows that there is a gradual transition between positive and negative APD-alt where there is no alternation (white areas in upper panel separate blue and red areas), although Ca-alt change abruptly from one myocyte to another (blue nodes next to red nodes).

Electrically discordant alternans are the most arrhythmogenic type, but concordant alternans often precede discordant alternans and play an important role. Ca-alt help in the identification of APD-alt, as they are concomitant, but also indicate dysfuntional contraction behavior. Discordant Ca-alt could aggravate the situation by increasing the number of myocytes that do not contract synchronously.

## Discussion

### Main findings

Mechanical and electrical alternans, which are known to be bidirectionally coupled, may compromise cardiac function. This study shows that alternans have their origin in Ca^2+^ alterations and reveals how Ca^2+^ impairment in HF or fibroblast interaction influences the development of cellular alternans and how they appear in cardiac tissue. Our main findings are as follows: (i) Ca^2+^ mechanisms, involving SERCA, I_CaL_, and NCX, are responsible for the occurrence of cellular alternans by modulating Ca^2+^ cycling to threshold levels; these mechanisms act differently in normal and failing mocytes, (ii) the degree of Ca^2+^ impairment, given by [Ca^2+^]_JSR_, determines the myocyte susceptibility to Ca-alt development, (iii) fibroblasts induce discordant Ca-alt in normal cardiac tissues, (iv) the high number of alternans in failing tissue with myocyte-fibroblast interaction is reduced due to exacerbated Ca^2+^ impairment, and (v) discordant Ca-alt are more prone to be translated into discordant APD-alt in failing tissue with fibroblasts than in normal tissue with fibroblasts. Fibroblasts and HF can therefore promote alternans because they alter Ca^2+^ dynamics, deteriorate contractility and increase the risk of cardiac arrhythmia. Restoring Ca^2+^ dynamics could thus potentially be used to prevent alternans and improve cellular contraction and cardiac function, although the differences between normal and failing tissues underline the need to consider HF when developing new pharmacological treatments.

### Origin of cellular alternans

Although cardiac alternans usually occur at fast pacing rates, the threshold for the onset of fluctuations can vary between individuals. It is also a characteristic outcome in heart disease. The first cellular theory for cardiac alternans was based on the relationship between APD alternans and the APD restitution slope [26]. Alternation was generated and sustained when the slope was higher than 1, so steeper restitution curves led to alternans at a longer PCL. However, we found that myocyte-fibroblast coupling, which shortens APD, promotes alternans at a longer PCL, despite a flatter restitution curve. These results indicate that there are other factors that contribute to generating alternans and that the analysis of the restitution curve has some limitations. If alternans are voltage-driven, as Xie et al. [45] found in the Phase I of the Luo and Rudy model [46], fibroblasts that shorten APD can induce alternans at a lower PCL. But when the electrophysiological model incorporates detailed Ca^2+^ cycling (as in our case) myocytes-fibroblast coupling promotes alternans because the alternans onset is then driven by Ca^2+^ cycling instabilities [45]. In fact, Ca^2+^ dynamics, which also oscillates from beat to beat, is one of the most important factors in alternans research. The bidirectional coupling between membrane voltage and intracellular Ca^2+^ explains why APD-alt and Ca-alt occur concomitantly [47], as can be seen in the restitution curves obtained for APD_90_ and Ca^2+^. However, there is still controversy about the origin and underlying causes of these fluctuations, due to the Ca^2+^ dependence of ion channels, which also control Ca^2+^ cycling [23,47–49].

In the present computational study, in which the ORd model was used [36], the alternans found in single-cell pairs and tissue are similar to the alternans in the initial isolated myocytes. The ORd model was validated to reproduce human APD alternans (around 10 ms) with beat-to-beat alternans in the Ca^2+^ subsystem at high frequencies (PCL< 300 ms). They also occur in a 100-endo-cell strand, and pacing rates faster than 230 ms cause 2:1 block [36], which agrees with our results in tissue. This type of conduction block can also be observed in cellular simulations, usually at fast frequencies, under conditions that prolong APs. The difference is that a second short AP is developed after an AP longer than the cycle length. In previous studies focusing on alternans mechanisms, these cases were considered as alternans because of their long-short pattern [50]. In tissue simulations, we found that the short APs developed in the externally stimulated myocytes did not propagate through the tissue because the myocytes were in a refractory period, leading to 2:1 block. Fig 2A shows that the long APD in failing myocytes causes loss of capture at higher PCLs and this explains why, at a fixed PCL of 300 ms, 2:1 block cases were only observed in HF tissue without fibroblasts. Despite the different mechanisms of alternans and the conduction block, both were included in the study due to their potential arrhythmogenic consequences.

Our first simulations at different PCLs determined that Ca-alt were easier to identify than APD-alt and so this was used as the criterion to detect cellular alternans. In previous studies, AP amplitude alternans have also been used to explain T-wave alternans instead of APD variations and were able to predict malignant arrhythmias in patients with systolic dysfunction [51]. However, the link between mechanical and electrical alternans has also proved that mechanical fluctuations are more visible than T-wave alternans, so that research on these factors could be useful for managing HF patients [21].

### Alternans mechanisms

Introducing natural electrophysiological variability into ionic channel conductances and transporters provides a population of coupled cells with different electrophysiological characteristics. The analysis of the model variants prone to alternans revealed the electrophysiological mechanisms that promote these fluctuations, i.e. the Ca^2+^-handling proteins (SERCA, I_CaL_, NCX, and J_rel_). This agrees with the findings of Zhou et al. [31] and Walmsley et al. [50] for non-failing single myocytes. Walmsley et al. [50] associated the appearance of alternans to APD prolongation in failing myocytes when I_Kr_ was low, but not to other parameters, since the alternans were measured as differences in AP and CaT duration. As a novelty, we introduced fibroblast coupling to normal and failing myocytes and then compared them to myocyte-myocyte coupling. The results indicated that although the mechanisms driving alternans in the different populations were similar they had opposite effects on normal conditions and HF.

CaMK appears to play an important role in alternans generation as it controls Ca^2+^ cycling [36,52]. This could partially explain the fact that HF enhances cellular alternans, since a 50% increase in the CaMK active fraction is a characteristic alteration of failing myocytes (Table 1). Ca^2+^-handling proteins help to maintain normal Ca^2+^ cycling in myocytes to ensure contractility and their disruption can lead to impaired Ca^2+^ dynamics in the different cell compartments. This has been linked to beat-to-beat Ca^2+^ oscillations [53], including fluctuations in the SR Ca^2+^ content [30], which are the root cause of cardiac alternans. Recent studies have reported that the SR imbalance between Ca^2+^ reuptake and Ca^2+^ release explains the genesis of Ca-alt [30,31]. We found that the SERCA pump, which uptakes Ca^2+^ into the sarcoplasmic reticulum (SR), is involved in alternans-prone myocytes, while SERCA overexpression may possibly suppress cardiac alternans, as has been shown by Cutler et al. [33]. However, unlike previous studies, in HF we found that alternans were not eliminated by increasing Ca^2+^ uptake into the SR [54–56]. Our results indicate that in HF, when CaT is strongly depressed alternans are not generated. However, if SERCA activity increases, Ca^2+^ cycling improves although it still remains at failure levels and can lead to alternans. These levels are mainly determined by junctional SR Ca^2+^ maximal content. Normal myocytes can also reach these failing Ca^2+^ levels when mechanisms like I_CaL_, SERCA, NCX, and I_NaK_ are altered, after which Ca-alt may occur. With reduced I_CaL_, Ca^2+^-induced Ca^2+^ release (CICR) tends to fail, high NCX contributes to intracellular Ca^2+^ imbalance by extruding Ca^2+^ out of the cell, and the higher I_NaK_ could indirectly facilitate inward NCX.

Many experimental studies have also highlighted the role of RyR in alternans generation due to RyR refractoriness and a failure in the CICR process [28,29,57]. Computer simulations with a spatially distributed Ca^2+^ cycling model integrated in the AP model could explain how Ca^2+^ sparks can lead to cellular alternans due to the randomness, refractoriness and recruitment properties of the couplon network [58]. However, in a less detailed Ca^2+^ cycling model, such as the one formulated in the ORd model, RyRs cannot show refractoriness, which could explain why we found that, unlike other parameters, changes in RyR hardly affected CaT. However, Tomek et al. [59] showed that slow dynamics and depressed SR Ca^2+^ release through RyR can also affect vulnerability to alternans. We therefore believe that incomplete JSR recovery is the factor that affects Ca^2+^ release and generates alternans [52].

### Alternans spatial distribution

Regarding the spatiotemporal patterns in cardiac tissue, we found that alternans entail different degrees of risk that depend on individual conditions. Electrical propagation runs smoothly in normal tissue, which explains the tissue’s spatial and electromechanical concordance. However, we found that cellular uncoupling in HF may promote the formation of alternans, in agreement with Hammer et al. [60], who found that myocytes with reduced intercellular coupling had higher alternans occurrence and amplitude, with a concordant pattern between neighboring cells. Fibroblasts also reduce intercellular coupling but can lead to spatially discordant alternans in normal tissue with fibroblasts, because, apart from reducing the CV they alter the myocytes’ electrical activity. Majumder et al. [61] observed discordant APD-alt preceding reentries in cultured monolayers of neonatal rat ventricular myocytes with Cx43 inactivation and myofibroblasts. Both slow CV and interstitial fibrosis were critical to the formation of such arrhythmogenic alternans at fast pacing. This agrees with our simulated HF and HF with fibroblast results, in which CV is depressed and discordant APD-alt are prone to occur. Despite the cellular uncoupling with fibroblasts we did not detect APD discordance in normal fibrotic fibers, but Ca-alt became spatially asynchronous due to the slow diffusion of Ca^2+^ ions [62] in comparison to the fast diffusion of membrane potential. As a consequence, cardiac tissue can present adjacent myocytes with both in-phase and out-of-phase V-Ca^2+^ coupling, which reduces APD-alt [63]. Electromechanically discordant alternans were also found by Xie et al. [45] when passive fibroblasts were inserted into a 2D tissue of rabbit ventricular myocytes. According to MacCannell et al. [38], the use of a passive fibroblast model hardly modulates myocyte AP or CaT, whereas the electrophysiological model does have a strong effect. As our objective was to analyze the most critical conditions only active fibroblasts were studied.

Electrophysiological remodeling of Ca^2+^-handling proteins is the main cause of Ca-alt in HF, which is then translated to APD-alt. Although we found spatially concordant alternans in HF tissue, they could become discordant at lower PCL, as was shown in [64]. There is also experimental evidence to show that HF enhances susceptibility to arrhythmogenic cardiac alternans, since ventricular fibrillation is only inducible after spatial discordance appears, which is more common in failing wedge preparations and occurs at a slower heart rate [54]. It is difficult to say whether or not these tissues included fibroblasts, but as optical mapping can be hampered by fibrotic areas, many experimental measurements avoid tissues with a high proportion of fibroblasts. In any case, of the computational alternans studies, this is the first to consider myocyte-fibroblast coupling in HF tissue. Apparently, fibroblasts do not increase discordance in failing tissues, since we did not find any differences with tissues without fibroblasts. Compared to normal tissue, HF involves more Ca^2+^ impairment and reduced CV, which may contribute to the positive APD-alt and Ca-alt coupling. Failing myocytes become susceptible to Ca-alt with unstable Ca^2+^ dynamics provided that CaT is not very depressed, and the resultant repolarization alternans can be high. If the tissue properties were to trigger spatial discordance, these alternans could become arrhythmogenic.

Arrhythmogenic activity in cardiac tissue can have a non-electric origin if Ca-alt is the initial cause. In contrast to normal hearts, the attempt to restore Ca^2+^ dynamics in HF could not improve cardiac function but did contribute to Ca-alt development and its associated effects. The conclusion is therefore that mechanisms that restore Ca^2+^ instabilities produced by fibroblasts and/or HF help to recover normal myocyte electrophysiological activity and avoid heart problems only if they are applied to specific heart conditions.

### Limitations

Mathematical models can help in the mechanistic analysis of alternans onset and complement experimental findings, although computational results may be limited by a number of uncertainties. Only one fibroblast model and one configuration for myocyte-fibroblast coupling were considered. The electrophysiological fibroblast model is thought to be more accurate, as it represents the ion currents in the fibroblasts, but using a different model (eg. passive fibroblast) or variations in the parameters could change the results. In our simulations in particular the fibroblast model does not take into account the potential Ca^2+^ cycling pathways in non-myocytes because of the lack of data related to cardiac fibroblasts, and this could contribute to Ca^2+^ dynamics in myocytes and therefore to developing alternans. Myofibroblasts, the contractile fibroblast activated form, arise in response to injury and present a different phenotype from fibroblasts. However, as no specific electrophysiological models of these contractile cells have been developed to date, in the present study we used the same mathematical fibroblast model in both normal conditions and HF, in spite of the fact that fibroblast electrophysiology may be altered in response to HF. We implemented the most critical of the different configurations for myocyte-fibroblast coupling, in which fibroblasts form conduction pathways and act as an electrotonic load. Representing fibroblasts as the same size as myocytes in a continuous mesh still has certain limitations.

Another limitation was the scarcity of human electrophysiological data, which would have been very useful to validate our findings. Our results were thus compared to animal models, including some species with a different electrophysiology to that of humans.

Despite its limitations, this study provides information that plausibly explains the response of myocytes to HF and fibroblast coupling in humans and can thus be used as a guide in the design of HF treatments.
